# Supplementary material for: Effects of dietary phosphates from organic and inorganic sources on parameters of phosphorus homeostasis in healthy adult dogs
Source: PLoS One. 2021 Feb 19;16(2):e0246950. doi: 10.1371/journal.pone.0246950 (PMC7894875; doi:10.1371/journal.pone.0246950)
Supplement: S1 Table — (DOCX) [file pone.0246950.s001.docx]

S1 Table: Serum calcium (sCa) concentrations [mmol/l] from pre- (t= 0) and up to 7 hours postprandially in adult healthy dogs fed a control (CON) and 3 high phosphorus diets, containing either poultry carcass meal (HPCM), NaH_2_PO_4_ (HPNaP) or KH_2_PO_4_ (HPKP) as a P source, for 18 days.

| sCa | | 0 | 0.5 | 1.0 | 1.5 | 2.0 | 3.0 | 5.0 | 7.0 |
| --- | --- | --- | --- | --- | --- | --- | --- | --- | --- |
|  |  | [h] | | | | | | | |
| CON | [mmol/l] | 2.5 ± 0.1 ^a^ | 2.5 ± 0.1 ^a^ | 2.6 ± 0.1 ^a^ | 2.6 ± 0.1 ^a,b^ | 2.5 ± 0.1 ^a^ | 2.6 ± 0.1 ^a,b^ | 2.6 ± 0.1 ^a^ | 2.6 ± 0.1 ^a^ |
| HPCM |  | 2.6 ± 0.1 ^a^ | 2.6 ± 0.1 ^a^ | 2.7 ± 0.1 ^a^ | 2.7 ± 0.1 ^b^ | 2.7 ± 0.1 ^a^ | 2.7 ± 0.1 ^a^ | 2.6 ± 0.1 ^a^ | 2.6 ± 0.1 ^a^ |
| HPNaP |  | 2.5 ± 0.1 ^a^ | 2.6 ± 0.1 ^a^ | 2.5 ± 0.1 ^a^ | 2.5 ± 0.1 ^a^ | 2.5 ± 0.2 ^a^ | 2.4 ± 0.1 ^b^ | 2.5 ± 0.5 ^a^ | 2.6 ± 0.1 ^a^ |
| HPKP |  | 2.5 ± 0.1 ^a^ | 2.7 ± 0.2 ^a^ | 2.8 ± 0.1 ^a^ | 2.7 ± 0.1 ^b^ | 2.7 ± 0.3 ^a^ | 2.7 ± 0.2 ^a,b^ | 2.6 ± 0.2 ^a^ | 2.6 ± 0.2 ^a^ |

| Reference range for healthy adult dogs: 2.3 – 3.0 mmol/l (Moritz, 2013). Values within one column, not sharing a superscript letter are significantly different (p<0.05). |
| --- |
