# Supplementary material for: Effects of dietary phosphates from organic and inorganic sources on parameters of phosphorus homeostasis in healthy adult dogs
Source: PLoS One. 2021 Feb 19;16(2):e0246950. doi: 10.1371/journal.pone.0246950 (PMC7894875; doi:10.1371/journal.pone.0246950)
Supplement: S2 Table — (DOCX) [file pone.0246950.s002.docx]

S2 Table: AUC_0-7_ for serum concentrations of minerals in adult dogs fed a control (CON) and 3 high phosphorus diets, containing either poultry carcass meal (HPCM), NaH_2_PO_4_ (HPNaP) or KH_2_PO_4_ (HPKP) as a P source, for 18 days.

| AUC_0-7_ | sP | sCa | sCaP | sNa | sK | sMg |
| --- | --- | --- | --- | --- | --- | --- |
|  | [mmol/l*t] | | [mg^2^/dl^2^*t] | [mmol/l*t] | | |
| CON | 9 ± 1 ^a^ | 17 ± 2 ^a,b^ | 25 ± 3 ^a^ | 1038 ± 30 ^a^ | 31 ± 1 ^a^ | 5 ± 1 ^a^ |
| HPCM | 10 ± 1 ^a^ | 18 ± 1 ^a^ | 25 ± 3 ^a^ | 1046 ± 9 ^a^ | 29 ± 1 ^a^ | 6 ± 0 ^b^ |
| HPNaP | 17 ± 3 ^b^ | 16 ± 1 ^b^ | 41 ± 5 ^b^ | 1064 ± 11 ^a,b^ | 29 ± 3 ^a^ | 5 ± 1 ^a,c^ |
| HPKP | 21 ± 3 ^c^ | 18 ± 3 ^a,b^ | 58 ± 4 ^c^ | 1102 ± 16 ^b^ | 35 ± 2 ^b^ | 5 ± 0 ^c^ |

| Values within one column, not sharing a superscript letter are significantly different (p<0.05). |
| --- |
