# Supplementary material for: Effects of dietary phosphates from organic and inorganic sources on parameters of phosphorus homeostasis in healthy adult dogs
Source: PLoS One. 2021 Feb 19;16(2):e0246950. doi: 10.1371/journal.pone.0246950 (PMC7894875; doi:10.1371/journal.pone.0246950)
Supplement: S3 Table — (DOCX) [file pone.0246950.s003.docx]

S3 Table: Serum parathyroid hormone (PTH) concentrations [ngl/l] from pre- (t= 0) and up to 7 hours postprandially in adult healthy dogs fed a control (CON) and 3 high phosphorus diets, containing either poultry carcass meal (HPCM), NaH_2_PO_4_ (HPNaP) or KH_2_PO_4_ (HPKP) as a P source, for 18 days.

| PTH | | 0 | 0.5 | 1.0 | 1.5 | 2.0 | 3.0 | 5.0 | 7.0 |
| --- | --- | --- | --- | --- | --- | --- | --- | --- | --- |
|  |  | [h] | | | | | | | |
| CON | [mmol/l] | 68 ± 24 ^a^ | 56 ± 16 ^a^ | 62 ± 17 ^a^ | 76 ± 30 ^a^ | 77 ± 31 ^a^ | 84 ± 35 ^a^ | 85 ± 35 ^a^ | 82 ± 33 ^a^ |
| HPCM |  | 68 ± 37 ^a^ | 65 ± 23 ^a^ | 84 ± 43 ^a^ | 111 ± 73 ^a^ | 90 ± 39 ^a^ | 61 ± 24 ^a^ | 88 ± 43 ^a^ | 52 ± 11 ^a,b^ |
| HPNaP |  | 55 ± 34 ^a^ | 120 ± 49 ^b^ | 174 ± 86 ^b^ | 237 ± 92 ^b^ | 241 ± 80 ^b^ | 289 ± 125 ^b^ | 164 ± 121 ^a,b^ | 50 ± 27 ^b^ |
| HPKP |  | 81 ± 38 ^a^ | 230 ± 152 ^a,b^ | 258 ± 164 ^a,b^ | 339 ± 252 ^a,b^ | 348 ± 171 ^b^ | 391 ± 197 ^b^ | 291 ± 154 ^b^ | 192 ± 131 ^a,b^ |

| Reference range for healthy adult dogs: 8- 45 ng/l (ALOMED laboratories). Values within one column, not sharing a superscript letter are significantly different (p<0.05). |
| --- |
