# Supplementary material for: Effects of dietary phosphates from organic and inorganic sources on parameters of phosphorus homeostasis in healthy adult dogs
Source: PLoS One. 2021 Feb 19;16(2):e0246950. doi: 10.1371/journal.pone.0246950 (PMC7894875; doi:10.1371/journal.pone.0246950)
Supplement: S4 Table — (DOCX) [file pone.0246950.s004.docx]

S4 Table: Serum crosslaps (CL) concentrations [ng/ml] from pre- (t= 0) and up to 7 hours postprandially in adult healthy dogs fed a control (CON) and 3 high phosphorus diets, containing either poultry carcass meal (HPCM), NaH_2_PO_4_ (HPNaP) or KH_2_PO_4_ (HPKP) as a P source, for 18 days.

| CL | | 0 | 0.5 | 1.0 | 1.5 | 2.0 | 3.0 | 5.0 | 7.0 |  |
| --- | --- | --- | --- | --- | --- | --- | --- | --- | --- | --- |
|  |  | [h] | | | | | | | | |
| CON | [ng/ml] | 0.3 ± 0.1 ^a^ | 0.3 ± 0.2 ^a,b^ | 0.3 ± 0.1 ^a,b^ | 0.2 ± 0.1 ^a^ | 0.2 ± 0.1 ^a^ | 0.2 ± 0.1 ^a^ | 0.2 ± 0.1 ^a^ | 0.2 ± 0.1 ^a^ |  |
| HPCM |  | 0.2 ± 0.1 ^a^ | 0.2 ± 0.1 ^a^ | 0.2 ± 0.1 ^a^ | 0.2 ± 0.0 ^a^ | 0.2 ± 0.1 ^a,b^ | 0.2 ± 0.1 ^a^ | 0.2 ± 0.0 ^a^ | 0.1 ± 0.1 ^a^ |  |
| HPNaP |  | 0.4 ± 0.1 ^a^ | 0.4 ± 0.1 ^b,c^ | 0.4 ± 0.2 ^b^ | 0.4 ± 0.1 ^a^ | 0.5 ± 0.2 ^b,c^ | 0.7 ± 0.2 ^b^ | 0.6 ± 0.2 ^b^ | 0.6 ± 0.4 ^a,b^ |  |
| HPKP |  | 0.5 ± 0.2 ^a^ | 0.5 ± 0.1 ^c^ | 0.8 ± 0.2 ^c^ | 0.7 ± 0.2 ^b^ | 0.5 ± 0.1 ^c^ | 1.1 ± 0.3 ^c^ | 1.1 ± 0.2 ^c^ | 0.9 ± 0.4 ^b^ |  |

Reference range for adult dogs: 0.11- 1.83 ng/ml (Belić et al., 2012). Values within one column, not sharing a superscript letter are significantly different (p<0.05).
