# Supplementary material for: Effects of dietary phosphates from organic and inorganic sources on parameters of phosphorus homeostasis in healthy adult dogs
Source: PLoS One. 2021 Feb 19;16(2):e0246950. doi: 10.1371/journal.pone.0246950 (PMC7894875; doi:10.1371/journal.pone.0246950)
Supplement: S5 Table — (DOCX) [file pone.0246950.s005.docx]

**S5 Table:** Serum bone alkaline phosphatase (bALP) concentrations [U/l] from pre- (t= 0) and up to 7 hours postprandially in adult healthy dogs fed a control (CON) and 3 high phosphorus diets, containing either poultry carcass meal (HPCM), NaH_2_PO_4_ (HPNaP) or KH_2_PO_4_ (HPKP) as a P source, for 18 days.

| bALP | | 0 | 0.5 | 1.0 | 1.5 | 2.0 | 3.0 | 5.0 | 7.0 |  |
| --- | --- | --- | --- | --- | --- | --- | --- | --- | --- | --- |
|  |  | [h] | | | | | | | | |
| CON | [U/l] | 9 ± 2 ^a^ | 8 ± 2 ^a^ | 8 ± 2 ^a^ | 8 ± 2 ^a^ | 8 ± 2 ^a^ | 9 ± 2 ^a,b^ | 8 ± 2 ^a^ | 8 ± 2 ^a^ |  |
| HPCM |  | 8 ± 2 ^a^ | 7 ± 2 ^a^ | 7 ± 1 ^a,b^ | 7 ± 1 ^a,c^ | 7 ± 1 ^a^ | 7 ± 2 ^a^ | 6 ± 2 ^a^ | 7 ± 1 ^a^ |  |
| HPNaP |  | 12 ± 4 ^a^ | 10 ± 4 ^a^ | 11 ± 4 ^a,b^ | 11 ± 4 ^b^ | 11 ± 4 ^a^ | 11 ± 5 ^a,b^ | 11 ± 5 ^a^ | 11 ± 4 ^a^ |  |
| HPKP |  | 14 ± 6 ^a^ | 13 ± 6 ^a^ | 14 ± 6 ^b^ | 13 ± 5 ^b,c^ | 14 ± 5 ^a^ | 14 ± 6 ^b^ | 12 ± 5 ^a^ | 13 ± 5 ^a^ |  |

| Reference range for adult dogs: 7.0 ± 2.5 U/L (2- 3 years old); 6.7± 3.6 U/L (3- 7 years old; Allen et al., 2000). Values within one column, not sharing a superscript letter are significantly different (p<0.05). |
| --- |
