# Supplementary material for: Effects of dietary phosphates from organic and inorganic sources on parameters of phosphorus homeostasis in healthy adult dogs
Source: PLoS One. 2021 Feb 19;16(2):e0246950. doi: 10.1371/journal.pone.0246950 (PMC7894875; doi:10.1371/journal.pone.0246950)
Supplement: S6 Table — (DOCX) [file pone.0246950.s006.docx]

S6 Table: Serum phosphorus (sP) concentrations [mmol/l] from pre- (t= 0) and up to 7 hours postprandially in adult healthy dogs fed a control (CON) and 3 high phosphorus diets, containing either poultry carcass meal (HPCM), NaH_2_PO_4_ (HPNaP) or KH_2_PO_4_ (HPKP) as a P source, for 18 days.

| sP | | 0 | 0.5 | 1.0 | 1.5 | 2.0 | 3.0 | 5.0 | 7.0 |  |
| --- | --- | --- | --- | --- | --- | --- | --- | --- | --- | --- |
|  |  | [h] | | | | | | | | |
| CON | [mmol/l] | 0.8 ± 0.3 ^a^ | 1.1 ± 0.1 ^a^ | 1.1 ± 0.2 ^a^ | 1.1 ± 0.3 ^a^ | 1.3 ± 0.4 ^a^ | 1.4 ± 0.2 ^a^ | 1.4 ± 0.2 ^a^ | 1.5 ± 0.2 ^a^ |  |
| HPCM |  | 1.0 ± 0.2 ^a^ | 0.9 ± 0.2 ^a^ | 1.0 ± 0.3 ^a^ | 1.0 ± 0.3 ^a^ | 1.0 ± 0.3 ^a^ | 1.3 ± 0.3 ^a^ | 1.7 ± 0.2 ^b^ | 1.8 ± 0.2 ^b^ |  |
| HPNaP |  | 0.8 ± 0.2 ^a^ | 1.1 ± 0.8 ^a^ | 2.2 ± 0.3 ^b^ | 2.4 ± 0.3 ^b^ | 1.9 ± 1.2 ^a,c^ | 3.2 ± 0.5 ^b^ | 2.5 ± 0.6 ^b^ | 1.9 ± 0.1 ^b^ |  |
| HPKP |  | 1.0 ± 0.2 ^a^ | 1.5 ± 0.3 ^a^ | 2.1 ± 0.3 ^b^ | 2.5 ± 0.7 ^b^ | 3.0 ± 0.5 ^b,c^ | 3.9 ± 0.7 ^b^ | 3.6 ± 0.6 ^c^ | 2.9 ± 0.8 ^b^ |  |

| Reference range for healthy adult dogs: 0.7 – 1.6 mmol/l (Moritz, 2013). Values within one column, not sharing a superscript letter are significantly different (p<0.05). |
| --- |
