# Supplementary material for: Effects of dietary phosphates from organic and inorganic sources on parameters of phosphorus homeostasis in healthy adult dogs
Source: PLoS One. 2021 Feb 19;16(2):e0246950. doi: 10.1371/journal.pone.0246950 (PMC7894875; doi:10.1371/journal.pone.0246950)
Supplement: S7 Table — (DOCX) [file pone.0246950.s007.docx]

S7 Table: Serum sodium (sNa) concentrations [mmol/l] from pre- (t= 0) and up to 7 hours postprandially in adult healthy dogs fed a control (CON) and 3 high phosphorus diets, containing either poultry carcass meal (HPCM), NaH_2_PO_4_ (HPNaP) or KH_2_PO_4_ (HPKP) as a P source, for 18 days.

| sNa | | 0 | 0.5 | 1.0 | 1.5 | 2.0 | 3.0 | 5.0 | 7.0 |
| --- | --- | --- | --- | --- | --- | --- | --- | --- | --- |
|  |  | [h] | | | | | | | |
| CON | [mmol/l] | 146.0 ± 1.1 ^a^ | 148.9 ± 2.2 ^a^ | 130.8 ± 5.9 ^a^ | 149.1 ± 1.2 ^a^ | 149.5 ± 1.0 ^a^ | 150.5 ± 1.5 ^a^ | 150.2 ± 1.6 ^a,b^ | 149.9 ± 1.8 ^a^ |
| HPCM |  | 148.9 ± 2.2 ^a^ | 150.5 ± 1.6 ^a^ | 150.1 ± 2.1 ^a^ | 150.5 ± 2.4 ^a,b^ | 151.2 ± 1.6 ^a^ | 149.6 ± 2.0 ^a^ | 148.4 ± 1.6 ^b^ | 148.4 ± 1.8 ^a^ |
| HPNaP |  | 148.1 ± 2.2 ^b^ | 151.6 ± 2.7 ^a^ | 151.4 ± 1.3 ^a^ | 152.0 ± 1.0 ^b^ | 152.8 ± 3.2 ^a^ | 151.7 ± 2.5 ^a^ | 152.3 ± 1.9 ^a,c^ | 134.7 ± 4.3 ^a,b^ |
| HPKP |  | 153.9 ± 1.9 ^a^ | 159.1 ± 5.4 ^a^ | 157.9 ± 5.5 ^a^ | 154.9 ± 1.5 ^c^ | 161.8 ± 7.1 ^a^ | 161.5 ± 11.5 ^a^ | 155.1 ± 1.4 ^c^ | 155.1 ± 2.8 ^b^ |

| Reference range for healthy adult dogs: 142- 151 mmol/l (Moritz, 2013). Values within one column, not sharing a superscript letter are significantly different (p<0.05). |
| --- |
