# Supplementary material for: Effects of dietary phosphates from organic and inorganic sources on parameters of phosphorus homeostasis in healthy adult dogs
Source: PLoS One. 2021 Feb 19;16(2):e0246950. doi: 10.1371/journal.pone.0246950 (PMC7894875; doi:10.1371/journal.pone.0246950)
Supplement: S9 Table — (DOCX) [file pone.0246950.s009.docx]

S9 Table: Serum calcium by phoshorus product (sCaP) concentrations [mg^2^/dl^2^] from pre- (t= 0) and up to 7 hours postprandially in adult healthy dogs fed a control (CON) and 3 high phosphorus diets, containing either poultry carcass meal (HPCM), NaH_2_PO_4_ (HPNaP) or KH_2_PO_4_ (HPKP) as a P source, for 18 days.

| sCaP | | 0 | 0.5 | 1.0 | 1.5 | 2.0 | 3.0 | 5.0 | 7.0 |
| --- | --- | --- | --- | --- | --- | --- | --- | --- | --- |
|  |  | [h] | | | | | | | |
| CON | [mg^2^/dl^2^] | 29.0 ± 4 ^a,b^ | 47.4 ± 38 ^a,b^ | 36.7 ± 6 ^a^ | 36.7 ± 6 ^a^ | 39.1 ± 8 ^a^ | 44.9 ± 6 ^a^ | 43.5 ± 7 ^a^ | 50.1 ± 9 ^a^ |
| HPCM |  | 31.3 ± 7 ^a^ | 29.0 ± 8 ^a^ | 32.3 ± 8 ^a^ | 33.9 ± 10 ^a^ | 34.1 ± 10 ^a^ | 41.4 ± 8 ^a^ | 56.5 ± 7 ^a^ | 58.3 ± 7 ^a^ |
| HPNaP |  | 24.0 ± 5 ^b^ | 47.8 ± 12 ^b^ | 69.7 ± 11 ^b^ | 75.8 ± 8 ^b^ | 78.2 ± 7 ^b^ | 94.5 ± 13 ^b^ | 76.1 ± 11 ^b^ | 60.9 ± 4 ^a^ |
| HPKP |  | 30.4 ± 5 ^a^ | 49.6 ± 9 ^b^ | 74.4 ± 13 ^b^ | 85.1 ± 22 ^b^ | 98.9 ± 19 ^b^ | 130.6 ± 30 ^c^ | 115.9 ± 12 ^c^ | 88.9 ± 20 ^b^ |

| Recommended threshold in adult dogs: 55 mg2/dl2 (Block et al., 1998). Values within one column, not sharing a superscript letter are significantly different (p<0.05). |
| --- |
