# Supplementary material for: Effects of dietary phosphates from organic and inorganic sources on parameters of phosphorus homeostasis in healthy adult dogs
Source: PLoS One. 2021 Feb 19;16(2):e0246950. doi: 10.1371/journal.pone.0246950 (PMC7894875; doi:10.1371/journal.pone.0246950)
Supplement: S10 Table — (DOCX) [file pone.0246950.s010.docx]

S10 Table: Serum fibroblast growth factor 23 (FGF23) concentrations [pg/ml] from pre- (t= 0) and up to 7 hours postprandially in adult healthy dogs fed a control (CON) and 3 high phosphorus diets, containing either poultry carcass meal (HPCM), NaH_2_PO_4_ (HPNaP) or KH_2_PO_4_ (HPKP) as a P source, for 18 days.

| sFGF23 | | 0 | 0.5 | 1.0 | 1.5 | 2.0 | 3.0 | 5.0 | 7.0 |
| --- | --- | --- | --- | --- | --- | --- | --- | --- | --- |
|  |  | [h] | | | | | | | |
| CON | [pg/ml] | 367 ± 115 ^a^ | 267 ± 29 ^a^ | 238 ± 40 ^a^ | 308 ± 145 ^a^ | 274 ± 130 ^a^ | 211 ± 106 ^a^ | 258 ± 104 ^a^ | 299 ± 100 ^a^ |
| HPCM |  | 296 ± 158 ^a^ | 359 ± 98 ^a,b^ | 275 ± 49 ^a,b^ | 282 ± 108 ^a^ | 284 ± 71 ^a^ | 288 ± 69 ^a,c^ | 266 ± 70 ^a^ | 280 ± 72 ^a^ |
| HPNaP |  | 760 ± 233 ^b^ | 662 ± 167 ^a,b^ | 700 ± 255 ^a,b^ | 760 ± 163 ^a,b^ | 550 ± 189 ^a,b^ | 683 ± 213 ^b,c^ | 532 ± 193 ^b^ | 575 ± 175 ^b^ |
| HPKP |  | 982 ± 388 ^b^ | 1060 ± 487 ^b^ | 1003 ± 435 ^b^ | 969 ± 450 ^b^ | 875 ± 332 ^b^ | 789 ± 297 ^b^ | 735 ± 242 ^c^ | 793 ± 297 ^b^ |

| Values for healthy dogs, according to literature (Harjes et al., 2017): 315 (211- 449) pg/ml. Reference range for humans: 10- 50 pg/ml (Yamazaki et al., 2002). Values within one column, not sharing a superscript letter are significantly different (p<0.05). |
| --- |
