# Supplementary material for: Effects of dietary phosphates from organic and inorganic sources on parameters of phosphorus homeostasis in healthy adult dogs
Source: PLoS One. 2021 Feb 19;16(2):e0246950. doi: 10.1371/journal.pone.0246950 (PMC7894875; doi:10.1371/journal.pone.0246950)
Supplement: S11 Table — (DOCX) [file pone.0246950.s011.docx]

S11 Table: AUC_0-7_ for the serum parameters parathyroid hormone (PTH), fibroblast growth factor 23 (FGF23), bone alkaline phosphatase (bALP) and crosslaps (CL) in adult dogs fed a control (CON) and 3 high phosphorus diets, containing either poultry carcass meal (HPCM), NaH_2_PO_4_ (HPNaP) or KH_2_PO_4_ (HPKP) as a P source, for 18 days.

| AUC_0-7_ | PTH | FGF23 | bALP | CL |
| --- | --- | --- | --- | --- |
|  | [ng/l*t] | [pg/ml*t] | [U/l*t] | [ng/ml*t] |
| CON | 550 ± 158 ^a^ | 1523 ± 429 ^a^ | 55 ± 12 ^a,b^ | 2 ± 1 ^a^ |
| HPCM | 533 ± 166 ^a^ | 1314 ± 734 ^a^ | 47 ± 10 ^a^ | 1 ± 0 ^a^ |
| HPNaP | 1271 ± 570 ^b^ | 3146 ± 1331 ^a^ | 79 ± 31 ^b^ | 4 ± 1 ^b^ |
| HPKP | 2056 ± 1032 ^c^ | 5863 ± 2209 ^b^ | 92 ± 37 ^a,b^ | 6 ± 1 ^c^ |

| Values within one column, not sharing a superscript letter are significantly different (p<0.05). |
| --- |
