# Supplementary material for: Effects of dietary phosphates from organic and inorganic sources on parameters of phosphorus homeostasis in healthy adult dogs
Source: PLoS One. 2021 Feb 19;16(2):e0246950. doi: 10.1371/journal.pone.0246950 (PMC7894875; doi:10.1371/journal.pone.0246950)
Supplement: Attachment — Submitted filename: Response to reviewers.docx [file pone.0246950.s012.docx]

We want to thank the editor as well as both reviewers for the interest, the positive feedback as well as the encouraging words!

The format was amended according to the Journal’s requirements.

Further comments Reviewer 2:

line 162 - the authors describe how Ca, Na, and K were measured, but how was serum P measured?

We indeed missed to name serum here. Now added

Could you also elaborate on blood sample handling procedures? i.e. how quickly was serum removed from cells? Were the samples screened for hemolysis (to rule out preanalytical artefact)

The following sentences were added: *Blood samples were allowed to clot for 20 minutes at room temperature before centrifugation (1.260 g) for 10 minutes and serum was aliquoted into several Eppendorf safe-lock cups. For FGF23 measurements, whole blood was allowed to clot for 2 hours before centrifugation at 560 g for 15 minutes. Preanalytical artefacts due to haemolytic samples can be ruled out*.

line 337 - In the legend for Fig 5, it may be helpful to define the acronyms for aD and HP for the reader

Thank you for the comment. The explanation for the abbreviations were added
line 392 - the sentence beginning with "Nevertheless..." is confusing, is the word "may" misplaced?

You are right. The sentence was corrected
line 443 - would improve readability by replacing "sol" with soluble

Thank you! Corrected
